# Effectiveness of Containment Measures to Control the Spread of COVID-19 in North Africa

**DOI:** 10.1017/dmp.2020.314

**Published:** 2020-09-03

**Authors:** Ouail Ouchetto, Asmaa Drissi Bourhanbour, Mounir Boumhamdi

**Affiliations:** LIMSAD-FSAC, Hassan II University of Casablanca, Casablanca, Morocco; FSJES-AC, Hassan II University of Casablanca, Casablanca, Morocco; Laboratory of Immunology, Ibn Rochd University Hospital Center, Casablanca, Morocco; Laboratory of Clinical Immunology and Immuno-Allergy, Faculty of Medicine and Pharmacy, Hassan II University of Casablanca, Casablanca, Morocco

**Keywords:** COVID-19, effective reproductive number, case of fatality rate, containment measures, North Africa

## Abstract

**Objectives:**

Since the first case of severe acute respiratory syndrome coronavirus-2, identified in December 2019, in Wuhan in China, the number of cases rapidly increased into a pandemic. Governments worldwide have adopted different strategies and measures to interrupt the transmission of coronavirus disease 2019 (COVID-19). The main objective was to report and evaluate the effectiveness of the adopted measures in North Africa countries.

**Methods:**

In these countries, the effective reproductive number R(t), the naïve case fatality rate, and the adjusted case fatality rate were estimated and compared on different dates.

**Results:**

The obtained results show that the early strict application of containment measures and confinement could help contain the spread of the epidemic and maintain the number of deaths low.

**Conclusions:**

These measures might be useful for other countries that are experiencing the start of local COVID-19 outbreaks. They could also serve to halt the spread of new epidemics or pandemics.

The World Health Organization (WHO) on March 11, 2020, declared the coronavirus disease 2019 (COVID-19) an outbreak a global pandemic. Indeed, since the first cases were reported in December 2019, in Wuhan in China,^1^ 210 countries and territories around the world have declared more than 4,500,000 confirmed cases and 300,000 deaths on 13 May, 2020. In a very short time, health-care systems and society have been severely challenged by this emerging virus. COVID-19 is an infectious disease caused by a newly discovered coronavirus. This pathogen was named severe acute respiratory syndrome coronavirus 2 (SARS-CoV-2).^2^ It is mainly transmitted by human-to-human contact (droplets and airborne). Preventing transmission and slowing the rate of new infections are the primary goals. The governments worldwide have adopted different strategies and measures to interrupt the transmission of COVID-19, such as containment measures. This term refers to efforts that aim to decrease or interrupt the transmission of COVID-19 in a population by minimizing physical contact between potentially infected individuals and healthy individuals. The basic reproductive number (R_0_) is used to measure the transmission potential of an epidemic.^3^ It represents the number of infections caused by an index case within a population with no pre-existing immunity. The value of R_0_ depends on the contact rate, transmission probability, and duration of infectiousness.^4^ R_0_ = 1 is considered a threshold for the development of an outbreak, referred to as the epidemic threshold.^5^ The time effective reproductive number R(t) represents the population-level transmission potential at time *t*.^6,7^ It allows tracking the evolution of transmission potential during an outbreak and when immunity intervention measures are implemented. It is also useful to study the impact of the adopted measures on the transmissibility of COVID-19 over time. The values of R(t) can also reveal the time when an outbreak was contained.

The North Africa countries announced different measures. In this study, we evaluate the effectiveness and impact of the adopted measures to contain the spread of COVID-19. We evaluate also the COVID-19 mortality rate on these countries by using the case fatality rate (CFR). We have excluded Libya from this study because there are very few confirmed cases and deaths reported. These figures may not reflect the actual COVID-19 data because of the conflict and the unstable political situation in Libya due to the civil war. In addition, the number of tests performed is very low and the contact tracing is also limited.

## METHODS

The data of the daily confirmed cases and number of deaths of COVID-19 were mainly obtained from the European Centre for Disease Prevention.^8^ For this study, we used the data from the first confirmed cases up to May 13, 2020.

### Estimation of Effective Reproductive Number

The R_0_ is fundamental to quantify the transmission potential of an epidemic in public health practice. However, R_0_ cannot reflect the time-varying nature of an epidemic. An R(t) can provide more information, because it tracks the subsequent evolution of transmission. For this, we use the Time-dependent (TD) method. It provides a good fit for the epidemic curve and calculates real-time effective reproduction number by averaging the overall transmission networks,^9^ and it uses a Bayesian statistical framework. In this method, the generation time (GT) is required and is defined as the interval between a case becoming infected and its subsequent infection of another case. We assume the GT is equal to the incubation period, and we suppose that TD is 5 d and the SD is equal to 1.9.^10^

The estimation of R(t) was performed by using the statistical package R0, which is coded in R language. In this package, we particularly used a function named ‘est.R0.TD’ to estimate R(t) according to Wallinga and Teunis, and the confidence interval is computed by multinomial simulations at each time step with the expected value of R(t).^9^ If R(t) > 1, the number of cases will increase, such as at the start of an epidemic; where R(t) < 1, there will be a decline in the number of cases.

### Estimation of CFR

The CRF is interpreted as the conditional probability of death. The naïve CFR (nCFR) is the first estimation, and it is defined as the ratio of the number of cumulative deaths D(t) to the number of cumulative confirmed cases C(t) at time t^11^:
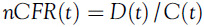



To obtain a more accurate estimation of CFR, we take into account the time interval [T] between case confirmation to death. The second estimation is the adjusted CFR (aCFR) and is defined as the ratio of the number of cumulative deaths D(t) at time t to the number of cumulative confirmed cases C(t-T) at time t-T^12^:
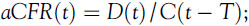



The mean time from illness onset to death was estimated at 20.2 d.^13^ For example, aCFR at t = 30 April is equal to the ratio of the number of cumulative deaths at t = 30 April to the number of cumulative confirmed cases at time t-T ≈ t-20, which is April 10.

## RESULTS

**FIGURE 1:**
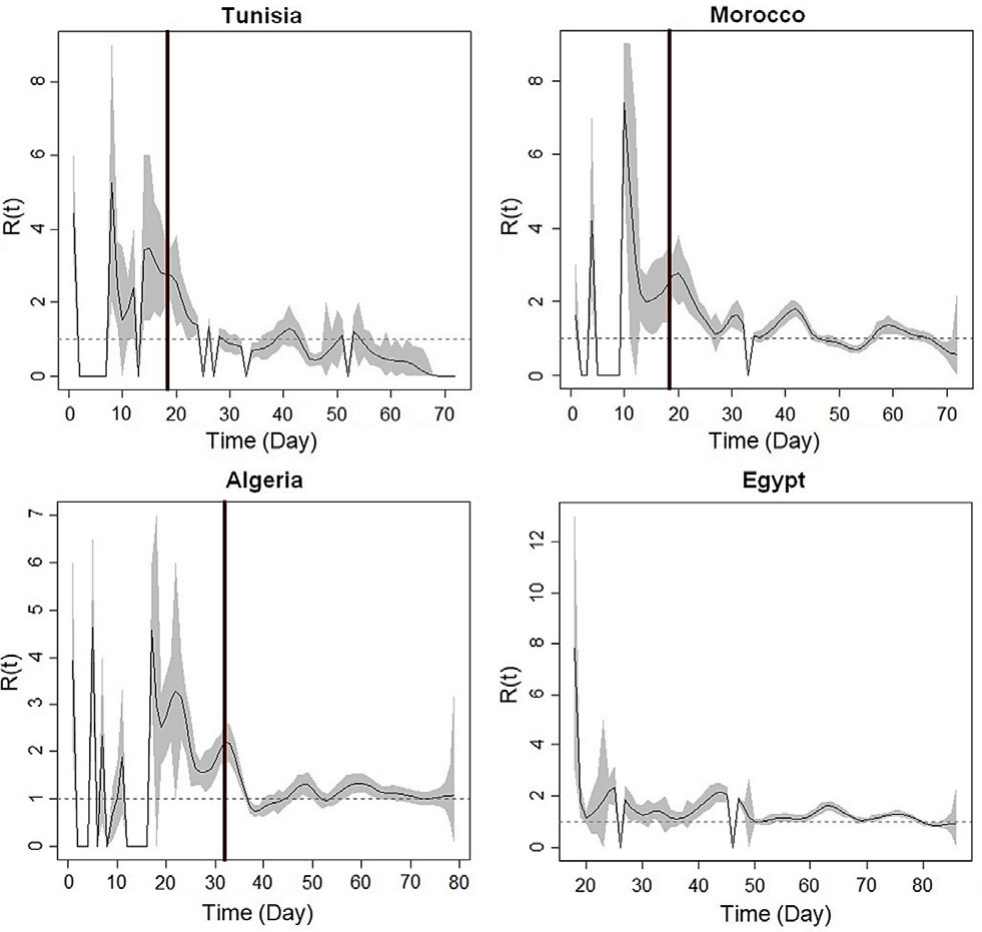
Real-Time Reproductive Number R(t) Values in the COVID-19 Outbreak in the Selected Countries (Morocco, Algeria, Tunisia, and Egypt), Calculated With TD Method. The black vertical line corresponds to the first day of confinement (T1).

The R(t) was computed for the studied countries from the date of the first confirmed cases until 13 May (Figure 1). On each graph, the shaded area corresponds to the 95% confidence interval, the black curve to the median, and the black vertical line to the first day of confinement (T1). At the beginning of the epidemic, we observe some peaks of R(t) that correspond to transmission clusters initiated by imported cases and have been controlled. These peaks can be ignored because they are limited to a few days. In the case of weekly incidences instead of daily ones, the curves would be much smoother. R(t) is null on some dates when zero cases were reported.

In the early phases, R(t) is higher than 3 for all countries. On T1, the value of R(t) was 2.76, 2.78, and 2.09 for Morocco, Tunisia, and Algeria, respectively. To show the effect of the introduced measures by each country, we compare the R(t) value 15 d after the confinement date (T2). R(t) value was 1.045 for Morocco, 0.684 for Tunisia, and 1.233 for Algeria. The R(t) values decreased in particular those of Tunisia, showing the effect of the introduced measures.

On May 13, the R(t) values of Tunisia and Morocco are significantly smaller compared with those of Egypt and Algeria. R(t) value was 0 for Tunisia, 0.545 for Morocco, 1.067 for Algeria, and 0.93 for Egypt. These data indicate that the outbreak is under control in Tunisia and Morocco.

**FIGURE 2:**
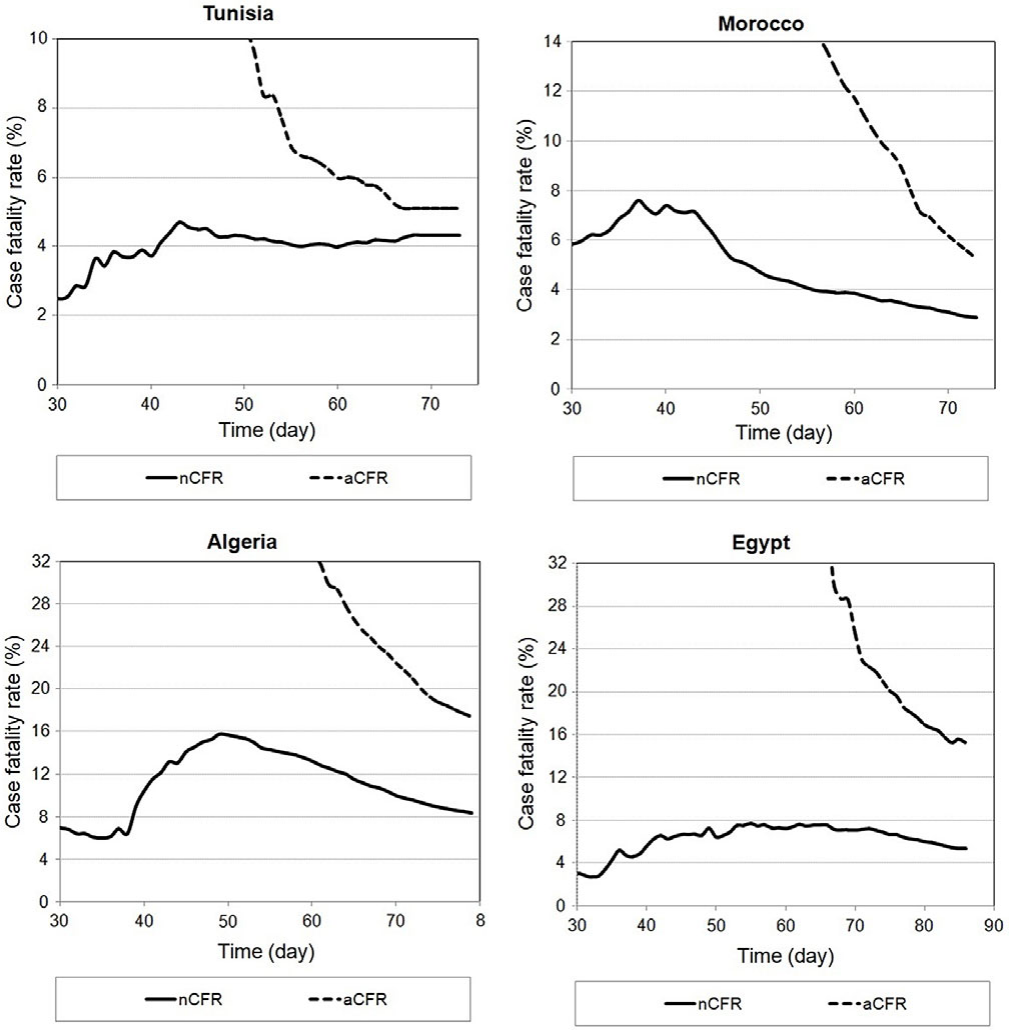
Case Fatality Rate (CFR) in the Selected Countries (Morocco, Algeria, Tunisia, and Egypt), Calculated With naïve CFR (nCFR) and adjusted CFR (aCFR) With T ≈ 20 d.

Until May 13, 2020, there were 45 deaths in Tunisia, 188 in Morocco, 522 in Algeria, and 565 in Egypt. The nCFR and aCFR values were estimated until 13 May (Figure 2). At the early stage, aCFR took considerably higher values than nCFR, and this is owing to the time interval T between case confirmation to death. For the 4 countries, the difference between aCFR and nCFR is decreasing during outbreak. For Tunisia, this difference is relatively constant in the last week, which is due to the stabilization of the number of cumulative deaths and the number of cumulative confirmed cases. The nCFR was 2.88% and aCFR was 5.26% for Morocco, nCFR was 4.31% and aCFR was 5.09% for Tunisia, nCFR was 8.21 % and aCFR was 16.91% for Algeria, and nCFR was 5.33% and aCFR was 15.19% for Egypt. The aCFR of Egypt and Algeria was high because of the high number of cumulative deaths.

## DISCUSSION

The first confirmed cases were reported on March 2, February 25, March 2, and February 14 in Morocco, Algeria, Tunisia, and Egypt, respectively. Confinement at home was imposed in Morocco and Tunisia on March 20 and in Algeria on March 28. Morocco, Algeria, and Tunisia were applied relatively the same measures but on different dates. In Egypt, the introduced measures were less stringent and confinement was not applied (Table 1).

**TABLE 1:** Adopted Containment Measures in Selected Countries (Morocco, Algeria, Tunisia, and Egypt)

	Morocco	Algeria	Tunisia	Egypt
Isolation of cases	Yes	Yes	Yes	Yes
Quarantine of contacts	Yes	Yes	Yes	Yes
Stay-at-home recommendations	Yes	Yes	Yes	No
Isolation of cities and regions	Yes	Yes	Yes	No
Total closure of leisure and catering area	Yes	Yes	Yes	No
Closure of educational institutions	Yes	Yes	Yes	Yes
Workplace closures	Yes	Yes	Yes	No
Mass gathering cancellations	Yes	Yes	Yes	Yes
Closure of places of worship	Yes	Yes	Yes	Yes
Closure of air borders	Yes	Yes	Yes	Yes
Closure of land borders	Yes	Yes	Yes	No
Closure of Sea borders	Yes	Yes	Yes	No
Obligation of face masks	Yes	No	No	No

At the beginning of the epidemic, the reproduction number R(t) of the 4 studied countries has high values, indicating that the outbreak of COVID-19 is in the propagation phase. Morocco and Tunisia applied the first measures of containment from March 13 (11 d after T0), and they imposed confinement at home in the early phases of the epidemic. For these reasons, on T1, the R(t) values were higher than those of Algeria. The effect of the measures and confinement takes time to manifest itself in the data, which is partly logical. A few days are needed for the symptoms to manifest, which creates a time lag.

On T2, the R(t) value of Tunisia and Morocco was lower than that of Algeria. This shows the impact of early confinement. Algeria started by applying restrictive measures and confinement of the Blida region only. It delayed imposing the confinement in the whole country (32 d after T0).

On May 13, R(t) value indicates that the COVID-19 decreased in Morocco and probably it is the beginning of the end of the epidemic in Tunisia. For Algeria and Egypt, R(t) values remained around 1, which indicate that the number of new cases was stable. An important thing to keep in mind when interpreting the results of R(t) is that this number depends heavily on the counts of laboratory-confirmed cases reported, which may not reflect the actual epidemic. Therefore, even if Egypt and Algeria have R(t) = 0.93 and R(t) = 1.067, respectively, their cumulative cases numbers are very low compared with those of the neighboring European countries, such as France, Spain, and Italy.^8^

The mortality rate is relatively high in Algeria and Egypt and low in Tunisia and Morocco (Figure 2). COVID-19 patients combined with high age, diabetes, obesity, or cardiovascular diseases were reported to have a higher risk of mortality. The prevalence of these risk factors is similar in the studied countries (Table 2).^14-17^ Therefore, the mortality rate difference can be attributed to the containment measures, which were applied early in Tunisia and Morocco. In Algeria and Egypt, continued monitoring of deaths caused by COVID-19 is required to better care for patients.

**TABLE 2:** Median Age, Diabetes, Obesity, and Cardiovascular Diseases (CVD) Prevalence in the Selected Countries (Morocco, Algeria, Tunisia, and Egypt)

	Morocco	Algeria	Tunisia	Egypt
Median age	28.9	27.8	32.4	23.8
Diabetes prevalence (%)	7	6.7	8.5	17.2
Obesity prevalence (%)	26.1	27.4	26.9	32
CVD prevalence cases per 100,000	8501 to 9500	6601to 7500	7501 to 8500	7501 to 8500

## CONCLUSIONS

To control the spread of COVID-19, the studied countries have chosen to adopt measures to avoid increased morbidity and, thereby, decrease the pressure on the health system. The early application of containment measures and confinement could help contain the spread of the epidemic and keep the number of deaths low. These measures might be useful for other countries that are experiencing the start of local COVID-19 outbreaks. They could also serve to inform evidence-based decisions by governments for future epidemics or pandemics to prevent the spread of new pathogens.
